# Patterns of Membrane Protein Clustering in Peripheral Lymphocytes as Predictors of Therapeutic Outcomes in Major Depressive Disorder

**DOI:** 10.3389/fphar.2019.00190

**Published:** 2019-03-12

**Authors:** Hector J. Caruncho, Tania Rivera-Baltanas, Raquel Romay-Tallon, Lisa E. Kalynchuk, Jose M. Olivares

**Affiliations:** ^1^ Division of Medical Sciences, University of Victoria, Victoria, BC, Canada; ^2^ Psychiatric Diseases Research Group, Galicia Sur Health Research Institute, Complexo Hospitalario Universitario de Vigo (CHUVI), SERGAS, CIBERSAM, Vigo, Spain

**Keywords:** biomarkers, depression, membrane protein clustering, therapeutic efficacy, antidepressants

## Abstract

There is an utmost necessity of developing novel biomarkers of depression that result in a more efficacious use of current antidepressant drugs. The present report reviews and discusses a recent series of experiments that focused on analysis of membrane protein clustering in peripheral lymphocytes as putative biomarkers of therapeutic efficacy for major depressive disorder. This review recapitulates how the ideas were originated, and the main findings demonstrated that analysis of serotonin transporter and serotonin 2 A receptor clustering in peripheral lymphocytes of naïve depression patients resulted in a discrimination of two subpopulations of depressed patients that showed a differential response upon 8 weeks of antidepressant treatment. The paper also reviews the usefulness of animal models of depression for an initial evaluation of membrane protein clustering in lymphocytes, which provides a screening tool to determine additional proteins to be further evaluated in depression patients. Finally, the present review provides a brief discussion of the general field of biomarkers of depression in relation to therapeutic outcomes and suggests additional ideas to provide extra value to the reviewed studies.

## Clinical Interest and General Approaches for Developing Novel Biomarkers of Depression

There is a pressing necessity to provide a better way for diagnosis, prognosis, and therapeutic treatment of psychiatric disorders. This is because in contrast to other medical specialties, psychiatry has lacked clear biological indicators (i.e., biomarkers) to help guide a proper diagnosis or prognosis or to ascertain the best therapeutic approach for individuals suffering mental disorders (for a general review of the neurobiology, physiopathology, and treatment of depression, see Otte et al., 2016). This is of particular interest in the context of major depressive disorder (MDD) when considering that, although antidepressants are clearly efficacious to treat MDD (Cipriani et al., 2018), a high percentage of patients fail to show a proper therapeutic response upon the first antidepressant treatment (Rush et al., 2006). Accordingly, there has been an exponential increase in the number of publications focusing on biomarkers of depression (almost 1,400 hits when searching for the term “biomarkers of depression” in the past 3 years in a recent PubMed search) and on biomarkers of antidepressant response (almost 200 hits when searching for the term “biomarkers of antidepressant response” in the past 3 years in a recent PubMed search). Although this blooming of publications indicates a keen interest by researchers in evaluating the efficacy of multiple biomarkers of depression, it seems that most of these biomarkers may not be specific for MDD, and at the same time, there is a need of additional studies and clinical trials to validate the efficacy of these putative biomarkers (Quevedo and Yatham, 2018).

Current approaches to the discovery of novel biomarkers of diagnosis and/or therapeutic efficacy for MDD are mostly based on technological advances in neuroimaging or on the use of “Omics” technologies (i.e., genomics or other “Omics” approaches primarily used to define peripheral biomarkers of MDD) (see Gururajan et al., 2016; Voegeli et al., 2017; Busch and Menke, 2018). Recently, a series of systematic reviews have evaluated the effectiveness of multiple biomarkers of depression using genomics (Menezes et al., 2019), epigenomics (Goud Alladi et al., 2018), metabolomics (MacDonald et al., 2018), antidepressant pharmacologic treatment response (Voegeli et al., 2017), inflammatory biomarkers (Smith et al., 2018; Yang et al., 2018), and neuroimaging biomarkers (Drago et al., 2018; Levy et al., 2019; Suh et al., 2019).

Hypotheses-based approaches can also help to define novel biomarkers of depression and complement the information obtained from neuroimaging and “Omics” studies, with the final purpose of finding specific combinations of biomarkers (including “Omics,” neuroimaging, and hypotheses-based approaches) that can be translated to the clinical and public health settings. In fact, some of the most replicated studies on biomarkers of depression are based on hypotheses-based approaches, such as alterations on serotonin transporter (SERT) binding in platelets or alterations in serum proinflammatory cytokines that may relate to specific inflammatory events underlying the pathophysiology of depression (reviewed in Gadad et al., 2018).

Following that line of thinking, during the past few years, we carried out an experimental approach to develop and test the hypothesis that alterations in the patterns of membrane protein clustering in peripheral lymphocytes can predict the therapeutic outcomes of psychopharmacological treatment in MDD. The present scientific review summarizes and discusses our findings, providing a proper context on how the studies were developed and points out toward additional experimental approaches designed for the validation and clinical translation of this approach.

## Development of the Hypothesis that Alterations in Membrane Protein Clustering may be a Putative Biomarker of MDD

During the second half of the 1990s, a series of reports from the laboratory of Drs. Erminio Costa and Alessandro Guidotti (University of Illinois at Chicago) provided the first demonstrations that the extracellular matrix protein reelin was heavily downregulated (about 50%) in multiple brain areas from schizophrenia post-mortem brain samples (Impagnatiello et al., 1998) and in the cerebral cortex of bipolar patients with psychotic episodes (Guidotti et al., 2000). These findings were followed by other laboratories that not only were able to replicate them but also demonstrated a downregulation of reelin levels in the hippocampus of schizophrenia, bipolar disorder, and major depression (Fatemi et al., 2000).

Reelin is an extracellular matrix protein involved in developmental regulation of neuronal migration and in regulation of neural plasticity in the adult brain, which (as mentioned above) is downregulated in multiple psychiatric disorders (see Ishii et al., 2016, as a review). Reelin primarily binds to the membrane receptors apolipoprotein receptor 2 and the very low density lipoprotein receptor in a heterodimeric combination and brings about the phosphorylation of the cytoplasmic adaptor protein DAB1, resulting in the activation of multiple signaling pathways resulting in the control of neural migration and cortical layer formation, promotion of protein translation, dendrite outgrowth and development of dendritic spines, and in regulation of glutamatergic synaptic plasticity (reviewed in Lee and D’Arcangelo, 2016).

From there on, studies focusing on the origin of reelin downregulation observed in psychiatric disorders (primarily in schizophrenia) pointed toward epigenetic alterations involving a hypermethylation of CpG islands in the reelin gene promoter as the cause of brain reelin deficits (recently reviewed in Guidotti et al., 2016). At the same time, studies on animal models of depression provided numerous evidences that reelin downregulation in the subgranular zone of the dentate gyrus may affect the maturation of dentate newborn neurons (Lussier et al., 2009, 2013a) and dysregulate the glutamatergic-GABAergic systems crosstalk in limbic brain areas (Lussier et al., 2013b). These alterations could be reversed by conventional antidepressants (Fenton et al., 2015) or by anti-inflammatory drugs with an antidepressant effect such as etanercept (Brymer et al., 2018). Reelin haploinsufficient heterozygous reeler mice show some subtle alterations in neurochemistry and behavior, but otherwise are almost indistinguishable from wild-type mice; however, we have found that these animals are extremely susceptible to the depressogenic effects of repeated subcutaneous injections of the stress hormone corticosterone and show depressive-like behavioral paradigms at corticosterone doses that fail to induce such behaviors in wild-type mice, which prompted us to consider reelin downregulation as a putative vulnerability factor for depression (Lussier et al., 2011). In summary, all these studies indicated a possible important role for reelin in the pathophysiology of depression (reviewed in Caruncho et al., 2016).

Concomitantly, other reports provided evidence indicating that reelin induces protein translation in synaptosome preparations (Dong et al., 2003), increases the number and clustering of synaptosomal membrane proteins (Caruncho et al., 2004), and promotes the clustering of the canonical reelin receptors ApoER2 and VLDLR (Strasser et al., 2004). For recent reviews of the canonical and non-canonical reelin signaling pathways, see Bock and May (2016) and Lee and D’Arcangelo (2016).

Reelin is also expressed in blood plasma (Smalheiser et al., 2000) where it is secreted by hepatocytes (Smalheiser et al., 2000) and platelets (Tseng et al., 2010), and plasma reelin levels are also altered in neuropsychiatric disorders (Fatemi et al., 2001).

## Alterations in Serotonin Transporter (SERT) and Serotonin Receptor 2A (5HT2A) Peripheral Lymphocytes from Animals with Reelin Deficits

The observations of reelin alterations in depression summarized above are the evidence that reelin induces membrane protein clustering (Dong et al., 2003; Caruncho et al., 2004), the finding that animals with reelin deficits (i.e., heterozygous reeler mice) are quite susceptible to the depressogenic effects of corticosterone (Lussier et al., 2011), and the demonstration of reelin expression in blood plasma and its alterations in psychiatric disorders (Smalheiser et al., 2000; Fatemi et al., 2001), prompted us to evaluate the possibility that animals expressing low reelin levels (such as heterozygous reeler mice) might show alterations in membrane protein clustering in peripheral blood cells.

We primarily centered our studies on analyzing the patterns of membrane clustering of two proteins pertaining serotonergic neurotransmission (SERT and 5HT2A) that are also expressed in lymphocytes and may be involved in the regulation of inflammatory processes (recently reviewed by Wu et al., 2018; also see Ahern, 2011). Alterations in SERT and 5HT2A are directly involved in the pathophysiology of depression and represent some of the targets of antidepressant medication, which is not surprising when considering the essential roles that the serotonergic system plays in the regulation of behavioral patterns directly affected in depression, such as mood, emotion, or sleep (for a recent review of the serotonergic hypothesis of depression, see Fakboury, 2016).

**Figure 1:**
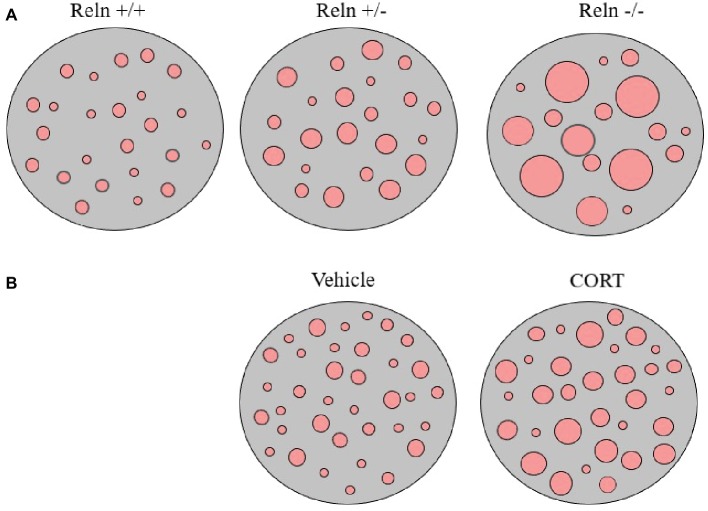
Schematic representation of serotonin transporter (SERT) clusters in the plasma membrane of one representative lymphocyte in reeler mice **(A)** and in rats treated with corticosterone **(B)**. Note that heterozygous reeler mice (Reln +/−) have an increase in SERT cluster number and size in comparison to wild-type mice (Reln +/+) and that homozygous reeler mice (Reln −/−) show much larger clusters that sometimes are difficult to differentiate [see Panel **(A)**, and Rivera-Baltanas et al., 2010]. After repeated-corticosterone treatment, a well-defined animal model of depression, SERT clusters also appear to increase in size [see Panel **(B)**, and Romay-Tallon et al., 2018].

Our studies analyzing the pattern of membrane clustering of the serotonin transporter protein (SERT) in peripheral lymphocytes from heterozygous reeler mice, null reeler mice, and wild-type mice showed a patchy pattern of expression of SERT immunolabeling in lymphocyte membranes that becomes disrupted in animals with reelin deficits (Rivera-Baltanas et al., 2010). In fact, heterozygous reeler mice showed a significant increase in SERT cluster size, while in null reeler mice SERT immunolabeling was mostly evidenced as a diffuse staining and was difficult to demonstrate well-detailed patches (Rivera-Baltanas et al., 2010). A schematic representation of alterations in SERT clustering in reeler mice lymphocytes is illustrated in Figure 1A.

Additionally, we also evaluated possibly alterations in SERT and 5HT2A protein clustering in the repeated-corticosterone model of depression (Romay-Tallon et al., 2018). This is a well-characterized animal model of depression (see Sterner and Kalynchuk, 2010, as a review) that we have used in many of our studies pertaining reelin and depression (see above). We found that repeated-corticosterone induced an increase in cluster size but not in cluster number for both SERT and 5HT2A (Romay-Tallon et al., 2018). A schematic representation of alterations in SERT clustering in lymphocytes from animals treated with corticosterone is illustrated in Figure 1B.

The results of these studies, together with evidence of alterations in SERT binding in lymphocytes of depression patients (Urbina et al., 1999; Lima and Urbina, 2002; Lima et al., 2005; Pena et al., 2005), brought us to develop the idea that perhaps the pattern of SERT clustering in lymphocytes might be disrupted in depression patients and if so to study if those alterations correlate with scores on psychological scales.

## Alterations in SERT and Serotonin 2A Receptor (5HT2A) in Peripheral Lymphocytes from Depression Patients

We hypothesized that in naïve depression patients (i.e., patients who had not taken antidepressants at least for several months) the pattern of SERT clustering in lymphocytes would follow the lines of that observed in heterozygous reeler mice (i.e., the average SERT cluster size would be larger in depression patients than in normal controls) and that response to antidepressant medication would be followed by a reversal of the alterations in the pattern of SERT clustering. Our findings generally showed that this was the case (i.e., naïve depression patients showed a similar number of SERT clusters per lymphocytes but they were of a larger size than those observed in samples from control non-psychiatric patients, for example, the average size of SERT clusters in the control population was about 0.11 and 0.14 μm^2^ for naïve depression patients). However, the analysis of the distribution of SERT cluster size allowed us to differentiate two subpopulations of naïve depression patients that we named D-I and D-II, the D-I subpopulation represented about ¾ of the patients and showed more than 40% of SERT clusters being between 0.05 and 0.010 μm^2^ (the modal peak of cluster size), while the DII subpopulation showed around 25% of SERT clusters between 0.05 and 0.010 μm^2^ (Rivera-Baltanas et al., 2012). Although we thought that these two subpopulations perhaps might reflect differential scores in psychological scales (i.e., in the Hamilton Depression Rating Scale, HDRS), when we proceeded to check the scores we observed that this was not the case, so that naïve depression D-I and D-II patients had similar HDRS scores (Rivera-Baltanas et al., 2012).

**Figure 2:**
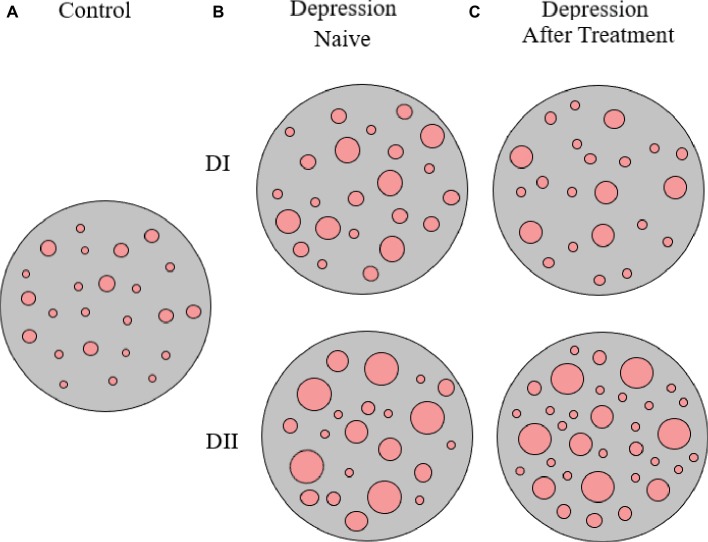
Schematic representation of SERT clusters in the membrane of lymphocytes from control **(A)** and depression patients **(B,C)**. Analysis of SERT clustering in naïve depression patients allowed the differentiation of two subpopulations of depression patients (D-I and D-II) that primarily differ in the average size of SERT clusters. After 8 weeks of antidepressant treatment, there were no changes in SERT cluster number and size for D-I patients, but there was an increase in the number of SERT clusters in D-II patients (for more details see text, and Rivera-Baltanas et al., 2012).

Interestingly, upon 8 weeks of psychopharmacological treatment, there was a differential response between D-I and D-II patients, as about half D-I patients showed no-response or a partial response to antidepressant medication, while the whole group of D-II patients responded to treatment and 75% of them showed remission of symptoms (see Table 1, and also Rivera-Baltanas et al., 2012, 2014, 2015). When analyzing the patterns of SERT clustering in lymphocytes after treatment, we found a significant increase in the number of SERT clusters within the size modal peak and a general increase in SERT cluster number in D-II patients, while there were no observable changes in the pattern of SERT clusters in D-I patients. A schematic representation of alterations in SERT clustering in lymphocytes from patients with depression is illustrated in Figure 2. In addition, the changes observed in D-II patients correlated with the amelioration of depression symptoms in these patients. These findings allowed us to suggest that analysis of the pattern of SERT protein clustering in lymphocytes could be considered a putative biomarker of therapeutic efficacy in MDD (Rivera-Baltanas et al., 2012) and prompted us to analyze the pattern of SERT clustering in relation to additional psychological scales like Self-Assessment Anhedonia Scale (SAAS) (Olivares et al., 2005; Rivera-Baltanas et al., 2015) and to study possible alterations in the pattern of clustering of other proteins like 5HT2A receptor (Rivera-Baltanas et al., 2014).

**Table 1:** Differential improvement in scores in the Hamilton Depression Rating Scale (HDRS) passed to D-I and D-II patients after 8 weeks of antidepressant treatment.

HDRS after treatment	Non-responders and partial responders (%)	Responders without remission (%)	Remission of symptoms (%)
Overall depression	36	32	32
D-I	45	33	22
D-II	0	25	75

Non-responders: HDRS scores improvement of less than 25%.

Partial responders: HDRS scores improvement between 25 and 50%.

Responders without remission: HDRS scores improvement of more than 50%, but absolute value of 7 or more.

Remission of symptoms: HDRS scores improvement of more than 50%, and absolute value below 7.

We focused on the study of the patterns of SERT clustering in depression patients in relation to a scale that measures anhedonia symptoms, because anhedonia is not only considered a key symptom of depression (APA, 2013) but is also conceptualized as a specific endophenotype of MDD (Pizzagalli, 2014) and a predictor of treatment response (Spijker et al., 2001). The use of the SAAS scale to provide a possible correlation between anhedonia scores and SERT clusters was also motivated by the issue that the HDRS does not properly evaluate anhedonia symptoms (Berrios and Olivares, 1995; Olivares and Berrios, 1998). When we evaluated the SAAS scores of D-I and D-II naïve depression patients, we did not found any difference between the two groups (Rivera-Baltanas et al., 2015). However, the post-treatment analysis evidenced differences in response between the D-I and D-II groups that were higher than those observed in the SERT-HDRS study, as the population of treated D-I patients did not improve at all SAAS scores, while D-II patients showed a remarkable improvement after 8 weeks of psychopharmacological treatment (Rivera-Baltanas et al., 2015). These findings may have some implications in developing proper strategies for the treatment of anhedonia symptoms in MDD, as anhedonia seems to be particularly refractory to treatment with current first-line antidepressant drugs (Shelton and Tomarken, 2001; Nutt et al., 2007; McCabe et al., 2009).

In a follow-up study, we studied possible alterations in the pattern on 5HT2A receptor clustering in lymphocytes in MDD (Rivera-Baltanas et al., 2014). We analyzed alterations in 5HT2A clustering in samples from a subset of the same population that we studied for SERT clustering (see above). We were surprised to find that measurements of 5HT2A clustering patterns in lymphocytes from naive MDD patients not only allowed us to again differentiate two patient subpopulations according to the distribution of 5HT2A cluster size, but that the same patients who were characterized as D-I and D-II when studying SERT clustering parameters (see above) were similarly shown as D-I or D-II when analyzing the characteristics of 5HT2A clusters and logically gave rise to a similar differential response to treatment (Rivera-Baltanas et al., 2014). In fact, these data made us think that perhaps there would be a general disturbance in membrane protein clustering in lymphocytes that may be operative in depression. Although logically additional studies on clustering patterns of other membrane proteins would be necessary to prove or falsify that hypothesis, we indicated that patterns of clustering of both SERT and 5HT2A receptor could be considered as putative biomarkers of therapeutic efficacy for MDD (Rivera-Baltanas et al., 2014).

As a possible limitation, we acknowledge that all these studies were carried out in a relatively small number of patients and thereby should be replicated in larger cohorts and also tested in a properly designed clinical trial.

## Additional Studies on Membrane Protein Clustering (MPC) in Lymphocytes in an Animal Model of Depression

In a very recent report, we evaluated the patterns of clustering of multiple proteins in the repeated-corticosterone model of depression (Romay-Tallon et al., 2018).

We centered our studies on proteins that tend to cluster into lipid rafts as alterations in G-protein-coupled receptor (GPCR) subunits integration into lipid rafts have been recently proposed as a putative mechanism of antidepressant actions (reviewed by Senese et al., 2018). As mentioned above, our analyses indicated that changes in SERT and 5HT2A receptor MPC in the repeated-corticosterone model of depression paralleled those observed in depression patients (Romay-Tallon et al., 2018). We also demonstrated that MPC patterns of SERT, 5HT2AR, dopamine transporter, and NMDA receptor 2B subunit, indicate an increase in cluster size but not in cluster number, while MPC analysis of beta-adrenergic receptor 2 gives rise to a decrease in receptor cluster size but no changes in numbers, and MPC study of pannexin 1 and prion cellular protein indicates that both the number and size of clusters are increased in the repeated-corticosterone model of depression (Romay-Tallon et al., 2018). Thereby, this study indicated the feasibility of using animal models of depression both to study alterations in MPC in lymphocytes (including the design of novel studies focusing on mechanistic approaches) and to screen for additional patterns of MPC to be further studied in MDD patients.

## Other Studies Designed to Facilitate Translation of MPC Studies to the Clinical Setting

Thinking about fostering the validation of MPC studies as a biomarker of therapeutic efficacy for MDD and how to bring those studies closer to the clinical setting, we came to the realization that most diagnoses of MDD are first determined by family physicians in a family clinic and that collection of samples for MPC studies will be much facilitated and also be cheaper if those analyses could be performed directly on blood smears instead that having a trained nurse drawing blood samples and then performing MPC studies on extracted lymphocytes as it was done for the studies mentioned in this review. Accordingly, we designed a comparative study of immunolabeling and analysis of MPC in extracted lymphocytes and in blood smears (Romay-Tallon et al., 2017) and were able to demonstrate that altering some parameters in fixation, incubation, and image analysis setting can result in similar measurements in MPC in whole blood drawn samples and in blood smears (Romay-Tallon et al., 2017). One should also consider that proper establishment of fixation, incubation temperature and time, and image analysis protocols are essential for an adequate standardization of MPC analysis technologies (for a more detailed discussion see Rivera-Baltanas et al., 2010 and Romay-Tallon et al., 2017).

## Additional Ideas and Experiments

The set of experiments discussed here clearly points to the interest of the MPC approach for developing of novel biomarkers of depression. Logically, this set of studies is complementary to the use of other technologies (i.e., “Omics,” neuroimaging, etc.) with the final intention of providing novel tools that result in a more precise approach to the use of the therapeutic arsenal for MDD treatment from a personalized medicine viewpoint.

We already mentioned the necessity of validating these studies in larger cohorts and in proper clinical trials. It is also a necessity to develop a way to perform automatic analyses of MPC that will facilitate the standardization for the technology and provide more accurate and faster measurements.

Next steps will logically involve the analysis in MPC patients of proteins that have been screened in animal models, as explained above, as this will also increase the efficacy of the test and the validation of the biomarker. It is also logic to think in using a similar approach for studies of other psychiatric disorders not only in terms of evaluating MPC as biomarkers of therapeutic efficacy but also of differential diagnoses.

Independently of research focusing on fostering MPC analyses as biomarkers of psychiatric disorders, there is also the necessity of developing mechanistic studies designed to evaluate how stress and depression bring about alterations in MPC in immune cells, what are the functional/pathological consequences of these alterations from a psychoneuroimmunology perspective, what are the mechanisms on antidepressant actions on MPC, and to evaluate if similar MPC alterations are also prevalent in the CNS and what would be the consequences of such changes. Finally, we can also surmise that these set of data may foster the idea of using MPC as a process to screen for antidepressant efficacy of novel compounds.

## Conclusions

The data discussed in this review indicate the feasibility of analysis of MPC as a technology to develop novel biomarkers of therapeutic efficacy for MDD, which hopefully together with other biomarker technologies can result in a more efficacious use of current antidepressant drugs.

## Author Contributions

HC wrote the initial draft of the review. All authors contributed to the original experiments and to the discussion, writing, and approval of the final version.

### Conflict of Interest Statement

The authors declare that the research was conducted in the absence of any commercial or financial relationships that could be construed as a potential conflict of interest.
